# Indications and Use of the Gluten Contamination Elimination Diet for Patients with Non-Responsive Celiac Disease

**DOI:** 10.3390/nu9101129

**Published:** 2017-10-18

**Authors:** Maureen M. Leonard, Pamela Cureton, Alessio Fasano

**Affiliations:** 1 Center for Celiac Research, Mucosal Immunology and Biology Research Center, Massachusetts General Hospital and Division of Pediatric Gastroenterology and Nutrition, Massachusetts General Hospital for Children, Boston, MA 02114, USA; pcureton@mgh.harvard.edu (P.C.); afasano@mgh.harvard.edu (A.F.); 2 Department of Pediatrics, University of Maryland School of Medicine, Baltimore, MD 21201, USA

**Keywords:** celiac, celiac disease, gluten, non-responsive, refractory, gluten contamination elimination, gluten-free diet

## Abstract

For the majority of patients diagnosed with celiac disease, once a gluten-free diet is initiated, symptoms improve within weeks and may completely resolve in months. However, up to 30% of patients may show signs, symptoms or persistent small intestinal damage after one year on a gluten-free diet. These patients require evaluation for other common GI etiologies and assessment of their celiac disease status in order to make a diagnosis and suggest treatment. Here, we propose an approach to evaluating patients with celiac disease with persistent symptoms, persistently elevated serology, and or persistent villous atrophy despite a gluten-free diet. We detail how to diagnose and distinguish between non-responsive and refractory celiac disease. Finally, we introduce the indications for use of the gluten contamination elimination diet and provide information for practitioners to implement the diet when necessary in their practice.

## 1. Introduction

Celiac disease (CD) is an autoimmune disease that develops in genetically predisposed individuals, triggered by the ingestion of gluten-containing grains. The development of accurate serological markers capable of screening individuals at risk of having CD has allowed the medical community to gain a better understanding of the epidemiology, heterogenous clinical presentation, pathophysiology, and management of the disease [1]. Once considered a rare gastrointestinal condition affecting young Caucasian children, CD is now recognized as a systemic autoimmune disorder that can develop at any age [2]. Diagnosis of CD is based on elevated serological auto-antibody studies, such as anti-tissue transglutaminase (anti-tTG), anti-endomysial antibody (anti-EMA), and anti-deamidated gliadin peptide (anti-DGP), and confirmed when biopsies of the small intestine obtained via esophagogastroduodenoscopy show histology consistent with villous blunting in addition to crypt hyperplasia and an increase in intraepithelial lymphocytes. After a patient is diagnosed with CD and has met with a dietitian to initiate the gluten-free diet (GFD), current guidelines endorse the measurement of serum auto-antibody levels every six months until normalization [3,4,5]. Once normalized, it has been presumed that mucosal recovery, secondary to dietary adherence, has occurred.

Upon initiating a GFD, the majority of patients diagnosed with CD may have an improvement in symptoms within weeks [6]. Patients are diagnosed with non-responsive CD if they have a recurrence or relapse of symptoms despite maintaining a GFD, and/or persistent villous atrophy despite adherence to a strict GFD after six to twelve months [7]. Up to 30% of patients with CD may show signs, symptoms or persistent villous atrophy or enteropathy after one year on a GFD and require evaluation [8,9,10,11]. Specifically, research shows that 25–40% of adults with CD have persistent enteropathy after two years on a GFD [8,12,13]. In adult patients with CD, persistent enteropathy is associated with the use of proton-pump inhibitors, non-steroidal anti-inflammatory drugs and selective serotonin uptake inhibitors [13]. Persistent enteropathy was also more common in older patients and males, and less common in patients with higher educational attainment [14]. In children, data suggests that 5–19% of patients with CD on a GFD may have persistent enteropathy despite treatment with a GFD for at least one year [9,10,15,16]. Additionally, characteristics such as the presence of symptoms at the time of the follow-up endoscopy, persistently elevated anti-tTG, and following a GFD for less than two years are not predictive of persistent enteropathy in children [10].

Patients with CD and persistent symptoms or enteropathy are described to have refractory CD if they have persistent villous atrophy despite adherence to a GFD, which can be due to a gluten-independent inappropriate activation of the immune system. The only effective treatment of these cases is the use of immunosuppressant therapy [17]. However, research suggests that in some cases, patients may not truly have refractory CD and instead may have signs or symptoms of persistently active disease in response to trace amounts of gluten, below the 20 parts per million threshold, which is considered safe for the majority of patients with CD [7]. In these cases, removal of any possible cross-contamination from the patient’s diet may result in remission and prevention of treatment with immunosuppressant agents [7]. These patients are diagnosed with non-responsive CD, rather than refractory CD. Differentiating between patients with non-responsive CD or a comorbid condition from patients with true refractory CD is an important distinction that must be made in order to initiate an effective treatment plan. This article aims to describe the evaluation of a patient with non-responsive CD, how to distinguish nonresponsive CD and refractory CD from each other and other comorbid conditions, and describe the indications for and how to implement the gluten contamination elimination diet.

## 2. Treatment with the GFD

For most patients diagnosed with CD, treatment with a GFD results in eventual improvement in symptoms, as well as a decrease in disease-associated serologic antibodies, and mucosal healing of the small intestine, also referred to as mucosal recovery [6,18,19]. While initial improvement may occur, maintaining a strict GFD can be difficult due to inadvertent cross-contamination during food preparation and processing and confusing food labeling. Compliance may be deterred due to palatability, social constraints and cost. In patients strictly following a GFD, even 1/100th of a slice of bread is enough to cause an immunogenic response, which can trigger signs and symptoms of CD over time [20]. Patients maintaining a GFD may still be inadvertently exposed to up to 2 g of gluten per day [21,22]. Additionally, despite an extremely strict regimen, patients with non-responsive CD have an incomplete response to the GFD. These patients may not be able to tolerate the trace amounts of gluten, less than 20 parts per million, that have been deemed safe for the majority of patients [7]. In comparison, patients with refractory CD lack a response to the GFD [12,18]. Thus, for this subset of patients, the GFD is insufficient to fully control the disease, and pharmacologic approaches are needed [7,17,23].

## 3. Evaluation of a Patient with Celiac Disease and Persistent Symptoms, Elevated Serology, and/or Persistent Enteropathy (Figure 1)

### 3.1. Evaluate Diagnosis of Celiac Disease

**Figure 1:**
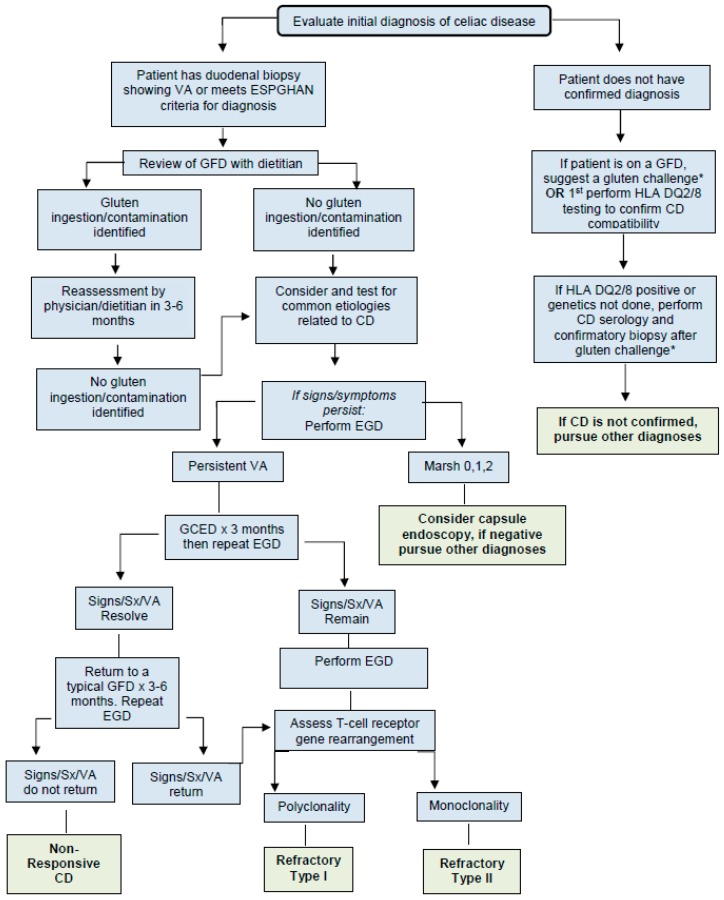
Evaluation of a patient with celiac disease and persistent symptoms, elevated serology, and/or persistent enteropathy. Abbreviations: VA: villous atrophy, ESPGHAN: European Society for Pediatric Gastroenterology Hepatology and Nutrition, GCED: gluten contamination elimination diet, GFD: gluten-free diet, HLA: human leukocyte antigen, CD: celiac disease, EGD: esophagogastroduodenoscopy, Sx: symptoms. * Gluten challenge: Current recommendations suggest a patient to eat approximately 3 g of gluten, which is equivalent to 1–2 slices of gluten-containing bread, daily for 2 to 6 weeks. Clinicians should consider a patient’s length of time on the GFD and symptomatic response to the challenge when determining the ultimate time course of the challenge [24].

Patients with signs or symptoms suggestive of non-responsive CD should first be evaluated by a gastroenterologist to ensure that the diagnosis of CD is correct and has been made accurately in accordance with Figure 1.

### 3.2. Evaluate for Gluten Contamination

Patients with CD presenting with new or persistent symptoms, and/or patients with persistently elevated serology despite a GFD, should first meet with a knowledgeable dietitian to assess their adherence to the GFD. A careful review of a patient’s diet, including medications, food preparation, and dining-out habits, often provides clues to possible gluten contamination that can contribute to the ongoing signs and symptoms. To identify possible sources of gluten ingestion, include the following in the discussion with the patient:

- Recheck labels of favorite everyday foods, as ingredients can change without notice;
- Contact manufacturers of products that contain the statement “manufactured in a plant that also produces or used on a machine that also processes wheat” to ask about the procedures they use to avoid cross-contamination;
- Recheck all over-the-counter and prescription medications with the manufacturers to be sure they do not contain gluten;
- Evaluate religious ceremony/holiday foods or communion hosts to be sure they contain less than 20 parts per million of gluten;
- Evaluate frequency and strategies used when dining away from home;
- Ensure any ingested oats are certified gluten free;
- Evaluate the tolerance of gluten-free oats in the diet. The addition of uncontaminated oats to the GFD has been tolerated by the majority of CD patients; however, a few people with CD may be clinically intolerant to oats [25];
- Look for sources of cross-contamination at home, and ensure the following are implemented in the home:
  ○ Use a separate toaster
  ○ Thoroughly clean kitchen counters
  ○ Use clean or separate cooking and serving utensils
  ○ Avoid “double dipping” in common condiment jars.

If any source of gluten contamination is identified, the patient should alter their current practice and return for reassessment by a dietitian and physician in three to six months. Once the possibility of cross-contamination has been ruled out, if symptoms and/or abnormal serology studies persist, other conditions and the CD status of the patient should be assessed to evaluate whether symptoms may be related to active CD or another ongoing process.

### 3.3. Consideration of Other Etiologies of Symptoms

Once gluten exposure of any kind has been excluded from the possible causes of persistent symptoms, elevated serology, and/or persistent enteropathy (if known), the physician should consider other etiologies that may be contributing to ongoing symptoms. The symptoms themselves will dictate the differential diagnosis. Conditions that may be considered based on symptoms include eosinophilic esophagitis, Crohn’s disease, autoimmune thyroid disease, infection, or other autoimmune diseases. Common etiologies of persistent symptoms in patients with CD are listed in Table 1.

### 3.4. Evaluation of Celiac Disease Status

Prior to the identification of accurate serology tests capable of screening patients for CD, patients underwent three small intestinal biopsies before a diagnosis of CD was confirmed. An initial esophagogastroduodenoscopy with small intestinal biopsies was completed when CD was suspected, a second was completed after a patient maintained a GFD to confirm remission, and a third esophagogastroduodenoscopy with small intestinal biopsies was completed after the patient was reintroduced to gluten to ensure that small intestinal damage returned with gluten consumption. The development of highly accurate diagnostic serological tests for CD also resulted in these serological tests being used to follow a patient’s adherence to the diet and as a marker of disease remission [26]. However, these tests have not been validated to measure dietary adherence or mucosal recovery in patients diagnosed with CD on a GFD, and their ability to predict these findings is poor [10,16,27,28,29]. For this reason, currently, the only way to confirm a patient with CD is in remission is by performing an esophagogastroduodenoscopy with biopsies to assess the histology of the small intestine.

## 4. Assessment and Treatment of Patients with Non-Responsive and Refractory Celiac Disease

Once gluten contamination and other possible conditions as the source of persistent symptoms or elevated serology have been excluded, patients should be evaluated for non-responsive CD, specifically refractory CD, by undergoing an esophagogastroduodenoscopy with biopsies. Patient with non-responsive CD and those with refractory CD type 1 present nearly identically, and may be distinguished based on their response to a diet that removes all possible sources of gluten contamination called the gluten contamination elimination diet. Patients with non-responsive CD or refractory CD type 1 typically, upon endoscopic assessment, have small intestinal villous atrophy, which upon further study has a normal intraepithelial immunophenotype and polyclonality of the T-cell gene receptor [17]. While at risk for complications due to persistent enteropathy, patients with refractory CD type 1 and non-responsive CD tend to have a benign course compared to those with refractory CD type 2. Patients with refractory CD type 2 are typically older at presentation and have more severe symptoms, which include diarrhea and weight loss. Refractory CD type 2 is defined by endoscopic evaluation that reveals enteropathy, an aberrant intraepithelial lymphocyte population and monoclonality of the T-cell gene receptor [17]. While rare, patients with refractory CD type 2 have a high risk of developing enteropathy associated T-cell lymphoma and have a five-year mortality rate of 50–60% [17,30]. While non-responsive CD often improves with dietary intervention, the only available treatment options for refractory CD are immunosuppressant agents, which have not been rigorously studied in CD and are often unsuccessful in cases of refractory CD type 2.

### 4.1. Immunosupressant Agents

Budesonide, a glucocorticoid, which acts locally in the intestine and has been studied in patients with refractory CD, is our first-line therapy for patients with refractory CD type 1 due to its low side-effect profile. While data is limited, a prospective, randomized pilot study found that patients treated with Budesonide and a GFD reported improved symptoms and higher overall well-being over a four-week period compared to the GFD alone [31]. Systemic glucocorticoids are rarely used for patients presenting with severe CD, such as those in celiac crisis.

Reports of patients with refractory CD responding to cyclosporine, azathioprine, and mesalamine compounds have been described in the literature [32,33,34]. The administration of Pentasa alone or in combination with Budesonide has been shown to improve symptoms in a small number of patients with refractory CD type 1 [34]. Additionally, several pro-inflammatory cytokines and lymphocyte recruiting chemokines, such as TNF-a, IFNy, IL-10, and IL-15, have been identified as crucial components in the inflammatory process, ultimately contributing to the small intestinal damage that occurs in patients with CD. Monoclonal antibodies to suppress pro-inflammatory cytokines are under investigation as possible therapeutic agents for the treatment of refractory CD. Further, cases describing the utility of Remicade, alemtuzumab, an anti-CD52 monoclonal antibody, and anti-IL15 antibodies have been reported [35,36,37,38].

### 4.2. Gluten Contamination Elimination Diet

The gluten contamination elimination diet was designed to eliminate any possible source of gluten exposure in the diet. In this way, it can serve to delineate patients with true refractory type 1 CD from patients with non-responsive CD. Patients with non-responsive CD may be responding to minute amounts of cross contamination in an already strict GFD and therefore the first step is to remove any possibility of gluten contamination. To achieve the elimination of any possible source of gluten in the diet, almost all processed foods, even those foods labeled gluten free, are removed; only whole, fresh unprocessed foods are allowed. Few exceptions are allowed to make the diet more palatable, such as vinegar, olive oil, and plain salt. The diet must be administered under the supervision of a knowledgeable dietitian to prevent unwanted weight loss or other nutritional deficiencies. Further, exceptions to the foods allowed and those that must be avoided can be made on an individual basis to help with compliance. Indications for the use of the gluten contamination elimination diet can be found in Table 2. Notably, we do not recommend its use for patients with newly diagnosed CD. Additionally, it is not advised or intended for patients with CD who have undergone a follow-up endoscopy after at least 12 months on a GFD and are found to have histology consistent with Marsh 0, Marsh 1, and in most cases Marsh 2, as we consider these findings suggestive of CD in remission. Recent literature in a small group of patients confirms that the gluten contamination elimination diet does not result in further histological improvement in these patients and is difficult to maintain given its restrictive nature [39].

#### 4.2.1. Initiation of the Gluten Contamination Elimination Diet

The first two weeks of the diet, Phase 1, will be the most restrictive on the gluten contamination elimination diet as the goal is to remove any gluten exposure from the diet in an effort to allow the immune system to go into remission (Table 3). Foods, such as dairy items, are removed to evaluate for lactose intolerance as a culprit for some of the symptoms present. After the first two weeks, Phase 2 of the gluten contamination elimination diet allows for the reintroduction of additional foods (Table 4). The gradual introduction of foods occurs over the next two to four weeks based on our experience. One new food is introduced at a time and added in every two-three days. If symptoms develop or worsen, the patient should consult with their dietitian and physician. Throughout the course of the diet, patients should document symptoms so that physicians can evaluate for improvement in certain areas, such as energy, frequency and severity of diarrhea, abdominal pain and headaches. In addition to the foods listed, a gluten-free multivitamin supplement should be taken daily. Any prescription medication should also be continued, if they have been confirmed to be gluten-free. Under the guidance of a dietitian, gluten-free products such as Ensure or Boost can be added to prevent unwanted weight loss.

#### 4.2.2. Duration of the Gluten Contamination Elimination Diet

It is recommended that the gluten contamination elimination diet be implemented for three months, and under the strict supervision of a knowledgeable dietitian. After completing the diet, the effectiveness of the diet is evaluated by repeating celiac serology and performing an esophagogastroduodenoscopy. If the previous serology was negative but histology was positive, a repeat esophagogastroduodenoscopy is the only way to determine if the diet has been successful. After completion of the diet, if symptoms persist but all measures are negative, then other conditions other than active CD must be considered.

#### 4.2.3. The Process of Returning to a Typical Gluten-free Diet

Due to the extremely restrictive nature of the gluten contamination elimination diet, it is not recommended for use for more than three to four months. Therefore, once the healing of the intestines has been confirmed via esophagogastroduodenoscopy, processed foods should be reintroduced into the diet. The rate of reintroduction of foods will be dictated by type of symptoms the patient had prior to the start of the diet. Those with elevated antibodies or villous atrophy but no symptoms may progress faster than those who are recovering from gastrointestinal symptoms or other complaints. Processed foods should be returned to the diet slowly to monitor for any reactions or symptoms that may develop. Foods that are the least likely to be contaminated with gluten should be added first; i.e., canned and frozen plain fruits, vegetables and meats, jarred sauces, condiments and salad dressing. If these foods do not produce any symptoms, the next step would be to reintroduce gluten-free grain products. In our experience, one serving of a gluten-free grain, such as one slice of gluten-free bread or 1/2 cup pasta or cereal, may be added each day for 3 days. If no symptoms appear, a second serving may be added. After the second serving of the processed gluten-free grain has been tolerated for 3–4 days, it would then be safe to return to a typical gluten-free diet.

The process of reintroduction should take about 2 weeks, depending on the individual tolerance. If symptoms occur at any time during the reintroduction, then the specific newly added item is removed until symptoms improve and reintroduced at a later date to confirm if that food was a problem. Again, the guidance of the dietitian is essential as the process should be individually tailored. Patients who have responded (with improvement of symptoms, serology, and/or histology) to the gluten contamination elimination diet should be re-evaluated after they have returned to a typical GFD for three to six months to ensure they are still in remission. In most cases, this will be through a careful intake of symptoms, and in most cases, repeat esophagogastroduodenoscopy [7]. If patients do not have a relapse of symptoms or villous atrophy upon return to a typical GFD they are in remission and should have routine care and follow-up with a gastroenterologist yearly. Patients who have are found to again develop signs or symptoms of active CD and/or villous atrophy on a typical GFD are evaluated and diagnosed with refractory CD and should be treated with immunosuppressant agents.

## 5. Conclusions

The increasing prevalence of CD, and of findings that persistent signs, symptoms and small intestinal enteropathy are more common in patients than previously thought, have stimulated a strong interest in identifying biomarkers of mucosal recovery and alternative treatments for patients with CD. The gluten contamination elimination diet is a useful tool to not only differentiate between patients with CD and those with true refractory CD, but also to treat patients with CD with persistent symptoms and villous atrophy despite adhering to a strict GFD. Given the restrictive nature of the gluten contamination elimination diet, it must be done under the supervision of a dietitian who can monitor the patient’s response and ensure nutritional adequacy while on this transitional diet. This is not recommended for patients recently diagnosed with CD, as it has not been shown to hasten mucosal recovery but has only been shown to improve clinical, serological, and histological measures in patients that have failed to respond to the GFD. Prospective studies are ultimately needed to evaluate the performance of this diet.

## Figures and Tables

**Table 1:** Etiologies of persistent symptoms in patients with CD.

Gluten Contamination
Change in fiber intake
Lactose Intolerance
Autoimmune enteropathy
Irritable bowel syndrome
Functional gastrointestinal disorders
Small-bowel bacterial overgrowth
Microscopic colitis
Pancreatic insufficiency
Refractory celiac disease

**Table 2:** Use of the gluten contamination elimination diet.

Indications for Use	Not for Use
Diagnosis of celiac disease is confirmed.	Diagnosis of celiac disease is not confirmed.
Patient has been on a gluten-free diet for 12 months.	Patient has non-celiac gluten sensitivity.
Patient has been seen by a dietician to review the diet for possible gluten exposure.	Patient has not been on a gluten-free diet for 12 months.
Patient has Marsh 3 damage on repeat small intestinal biopsy with or without elevated celiac antibodies.	Patients has Marsh 2 damage on repeat endoscopy in the presence of normal serology and no signs or symptoms associated with CD.
Proper education, support, and follow-up can be provided over the next 3 months.	Patient has Marsh 0-1 damage on repeat small intestinal biopsy with or without elevated celiac antibodies.
May consider use on a case by case basis for patients with persistent symptoms, elevated serology, and Marsh 2 damage on repeat small intestinal biopsy.	

**Table 3:** The Gluten Contamination Elimination Diet: Phase 1.

Phase 1: Week 1–2	
*Foods Allowed*	
Fruits/Vegetables	All Fresh Fruits and Vegetables (no frozen or canned fruits/vegetables)
Grains	Rice (brown and white) (preferably labeled gluten free)
Proteins	Chicken Turkey (not self-basting) Fresh Fish/seafood Eggs
Beverages/Nutritional Supplements	100% Fruit/Vegetable Juices Gatorade Fresh ground coffee 100% black or green tea (no herbal teas) Boost, Ensure (no malt flavor)
Seasoning/Condiments/Misc.	Fresh herbs (no dried herbs) Salt Fresh ground pepper Plain honey Olive oil Vinegar (excluding malt vinegar and flavored vinegars)

**Table 4:** The Gluten Contamination Elimination Diet: Phase 2.

Phase 2: Week 3–12	
Gradually Introduce Any of the Following Items over the Next Few Weeks. One New Food Can Be Introduced at a Time, With a New Food Added Every 2–3 Days. If Symptoms Develop or Worsen, Consult with Your Dietitian/Physician	
*Foods Allowed*	
Dairy (if tolerated)	Butter Yogurt (plain, unflavored) Cream (plain, unflavored) Cheeses Only ingredients: pasteurized milk, cheese cultures, salt, enzymes Cottage cheese Only ingredients: cultured milk, cream, and salt
Fruits/Vegetables	All Fresh Fruits and Vegetables (no frozen or canned fruits/vegetables)
Grains	Dried beans Carefully clean and sort for foreign particles Rice (brown and white) (preferably labeled gluten free)
Proteins (fresh only)	Chicken Turkey (not self-basting) Fresh fish/seafood Beef Pork and Lamb (no ham or bacon) Nuts in the shell or raw Eggs
Beverages/Nutritional Supplements	100% Fruit/Vegetable Juices Gatorade Fresh ground coffee 100% black or green tea (no herbal teas) Boost, Ensure (no malt flavor)
Seasoning/Condiments/Misc.	Fresh herbs (no dried herbs) Salt and fresh ground pepper Plain honey and sugar Lemon Olive oil Vinegar (excluding malt vinegar and flavored vinegars)
